# Protein Hydrolysate Stimulates Growth in Tomato Coupled With N-Dependent Gene Expression Involved in N Assimilation

**DOI:** 10.3389/fpls.2018.01233

**Published:** 2018-08-22

**Authors:** Francesco Sestili, Youssef Rouphael, Mariateresa Cardarelli, Anna Pucci, Paolo Bonini, Renaud Canaguier, Giuseppe Colla

**Affiliations:** ^1^Department of Agricultural and Forestry Sciences, University of Tuscia, Viterbo, Italy; ^2^Department of Agricultural Sciences, University of Naples Federico II, Portici, Italy; ^3^Consiglio per la Ricerca in Agricoltura e l’Analisi dell’Economia Agraria, Centro di Ricerca Orticoltura e Florovivaismo, Pontecagnano, Italy; ^4^NGA Laboratory, Tarragona, Spain; ^5^Nixe, Valbonne, France

**Keywords:** ammonium and nitrate transporters, biostimulants, N metabolism, amino acids, peptides, substrate drench application, *Solanum lycopersicum* L.

## Abstract

Plant-derived protein hydrolysates (PHs) have received increased attention in the last decade because of their potential to improve yield, nutritional quality as well as tolerance to abiotic stressors. The current study investigated the effects and the molecular mechanisms of a legume-derived PH under optimal and sub-optimal nitrogen (N) concentrations (112 and 7 mg L^−1^, respectively) in tomato (*Solanum lycopersicum* L.). Growth and mineral composition of tomato plants treated with PHs by foliar spray or substrate drench were compared to untreated plants. In addition, the expression was determined of genes encoding ammonium and nitrate transporters and seven enzymes involved in N metabolism: nitrate reductase (*NR*), nitrite reductase (*NiR*), glutamine synthetase 1 (*GS1*), glutamine synthetase 2 (*GS2*), ferredoxin-dependent glutamate synthase (*GLT*), NADH-dependent glutamate synthase (*GLS*), and glutamate dehydrogenase (*GDH*). The root and total plant dry weight, SPAD index and leaf nitrogen content were higher by 21, 17, 7, and 6%, respectively, in plants treated by a substrate drench in comparison to untreated tomato plants, whereas foliar application of PH gave intermediate values. PH-treated plants grown with lower N availability showed reduced expression of *NR* and *NiR* as well as of nitrate and ammonium transporter transcripts in both leaf and root tissues in comparison with untreated plants; this was especially pronounced after application of PH by substrate drench. Conversely, the transcript level of an amino acid transporter gene was up-regulated in comparison with untreated plants. At high N regime, the transcript levels of the ammonium and amino acid transporters and also *NR*, *NiR*, and *GLT* were significantly up-regulated in root after PH foliar and substrate drench applications compared with untreated plants. An up-regulation was also observed for *GS1*, *GS2*, and *GDH* transcripts in leaf after substrate drench. These results highlighted the potential benefits of using legume PH in vegetable production systems to increase growth and N-nutritional status of plants.

## Introduction

Nitrate (NO_3_^−^) constitutes the most important and available form of nitrogen (N) taken up readily in large quantities by vegetable crops to secure maximal productivity (Colla et al., 2010, 2011). However, the high production cost of N fertilizers as well as its high mobility and facility to leach into groundwater made N use a major environmental threat throughout the world (Tilman et al., 2002).

Any improvement in crop management practices that increases N capture efficiency should reduce the environmental pollution without affecting the reliability and stability of agricultural crop yield (Jannin et al., 2013; Santi et al., 2017). Many attempts have been proposed to enhance N use efficiency in vegetables by means of traditional breeding programs and genetic engineering; however, the commercial success of these cultivars has been very limited (Colla et al., 2010, 2011). More recently, the use of plant biostimulants which include beneficial microorganisms (i.e., mycorrhizal fungi and plant growth promoting rhizobacteria) as well as natural substances or compounds (i.e., humic acids, seaweed extracts, and protein hydrolysates) has been introduced as an efficient, safe and environmentally friendly approach to ensure high yield and improve the quality in a sustainable manner (i.e., by enhancing nutrient use efficiency) (Colla and Rouphael, 2015; du Jardin, 2015; Rouphael et al., 2015, 2017c; Colla et al., 2017b).

Plant-derived protein hydrolysates (PHs) have gained prominence globally as natural plant biostimulants in vegetable cropping systems (Ertani et al., 2014, 2015; Colla et al., 2015). Plant-derived PHs are mainly produced by enzymatic hydrolysis of plant biomass such as legume seeds, alfalfa hay, and plant by-products (Ertani et al., 2013; Colla et al., 2015, 2017b). Particularly, PHs coming from agricultural organic waste are gaining interest among the biostimulant enterprises and scientists, since they could be considered an efficient solution to the problem of plant by-product disposal, turning them into economic benefits for the growers (Pecha et al., 2012; Santi et al., 2017). Plant-derived PHs are a source of free amino acids and soluble peptides, and can also contain carbohydrates, phenols and limited amounts of plant nutrients (Calvo et al., 2014; Colla et al., 2015). Foliar or root applications of plant-derived PHs may activate several molecular and physiological mechanisms, in a wide range of horticultural commodities, that stimulate seedling and plant growth (Colla et al., 2014; Amirkhani et al., 2016), improve yield and nutritional quality (Colla et al., 2017a; Rouphael et al., 2017b) and mitigate the impact of a wide range of abiotic stresses such as salinity (Lucini et al., 2015), alkalinity (Rouphael et al., 2017a), and thermal stress (Botta, 2013). Recent review papers (Calvo et al., 2014; Halpern et al., 2015; Colla et al., 2015, 2017b) aiming to elucidate the mechanisms regulating these positive effects indicate that these products could affect crops by stimulating N metabolism through the regulation of key enzymes involved in N assimilation, and interfering with hormone-like activity (Schiavon et al., 2008; Ertani et al., 2009; Colla et al., 2014). PHs have been also shown to modulate the crop root system architecture (in particular the number of lateral roots), thus affecting the efficiency and uptake with which PH-treated plants explore the soil and capture nutrients (Ertani et al., 2013; Colla et al., 2015, 2017b; Nardi et al., 2016).

Inorganic N is absorbed by the roots of higher plants and it is rapidly turned into ammonium through the coordinated action of two key enzymes (Nitrate Reductase-NR and Nitrite Reductase-NiR). The first enzyme is the limiting factor of nitrate assimilation as it reduces nitrate in nitrite that is the substrate for the next reaction catalyzed by NiR that leads to the production of ammonium. This latter is then incorporated into glutamine by glutamine synthetase (GS). This step is a crucial checkpoint of plant growth, as it allows the first incorporation of the mineral nitrogen (Hirel et al., 2007). Two GS isozymes (cytosolic GS1, and plastidic GS2) have been identified in higher plants (Bernard and Habash, 2009). Their different organ- and cell-specific expression suggests a distinct function. GS1 is located usually in the cytosol of vascular tissues, involved in N recycling, and plays also a role in N mobilization in germinating kernels (Zhang et al., 2017). GS2 is mainly expressed in leaf mesophyll and re-assimilates the ammonium released during the process of photorespiration (amino acid turnover) or nitrate reduction (Husted et al., 2002).

The enzyme glutamate synthases (GOGATs) catalyze the conversion of glutamine and 2-oxoglutarate to glutamic acid that is nitrogen donor to other amino acids in subsequent transamination reactions (Bernard and Habash, 2009). Two forms of GOGAT, ferredoxin- (Fd-GOGAT or GLT), and NADH-dependent (NADH-GOGAT or GLS), have been identified in higher plants. The first is the predominant enzyme for glutamate synthesis in photosynthetic tissues; GLS is the major enzyme in non-photosynthetic tissues (Lancien et al., 2007). Glutamate dehydrogenase (GDH) is an important branch-point enzyme between carbon and nitrogen metabolism, because it catalyzes the reversible oxidative deamination of glutamate to 2-oxoglutarate and ammonia (Tercé-Laforgue et al., 2013).

Although the stimulation of NO_3_^−^ assimilation enzymes (NR, NiR, GS, and GOCAT) in both leaf and root tissues of maize seedlings after the application of an alfalfa-PH has been documented (Schiavon et al., 2008); however, the molecular mechanism(s) that may elucidate the mode of action of commercial legume-derived PHs under sub-optimal N conditions remain unknown.

It is well established that root systems respond to N limitation in the soil solution by two important adaptive responses (i) up-regulation of the high-affinity transport system (HATS) for NO_3_^−^ (<0.5 mM of external nitrate) and (ii) stimulation of lateral root growth (Remans et al., 2006). In their work, Remans et al. (2006) demonstrated that high-affinity nitrate transporter 2.1 (NRT2.1) plays a key role in the coordination of the root development, acting on lateral root initiation under low nitrate regime; whereas high-affinity nitrate transporter 2.3 (NRT2.3) was involved in the root-to-shoot long distance transport and nitrate uptake (Fu et al., 2015). Because PHs contain amino acids and peptides, the expression of a key gene encoding the amino acid transporter AAT1 (previously named Solyc11g0084401.1; Snowden et al., 2015) could provide further insight into the effects of PHs on amino acid turnover and allocation.

Based on these considerations, the aim of the current study was to assess the morphological, compositional and molecular changes in tomato plants grown under optimal and sub-optimal N conditions in response to PH application (foliar spray or substrate drench) in order to unravel the molecular mechanisms that may elucidate its mode of action.

## Materials and Methods

### Plant Material and Growth Conditions

The experiment was conducted in the 2015 summer growing season in a polyethylene greenhouse at the experimental farm of Tuscia University (latitude 42° 25′ N, longitude 12° 08′, altitude 310 m). The tomato (*Solanum lycopersicum* L. cv. Console F1, Società Agricola Italiana Sementi-SAIS, Cesena, Italy) seedlings were transplanted on June 8 at the four true leaf stage into plastic pots (diameter 14 cm and height 12 cm) containing 1.5 L of quarziferous sand with a particle size between 0.4 to 0.8 mm. Plastic pots were arranged in single rows on 16 cm wide and 5 m-long troughs at a plant density of 11 plants m^−2^ (30 cm between pots and 30 cm between troughs). The daily air temperature inside the greenhouse was maintained between 18 and 30°C by forced ventilation and day/night air relative humidity was 55/85%.

### Treatments, Experimental Design, and Nutrient Solution Management

Six treatments were compared, which derived by the factorial combination of two N levels in the nutrient solution (low, 7 mg L^−1^; high, 112 mg L^−1^) and three biostimulant application treatments (untreated, foliar spray, or substrate drench). The treatments were arranged in a randomized complete-block design with three replications per treatment, amounting to a total of 18 experimental plots with 15 plants each.

The commercial legume-derived protein hydrolysate Trainer^®^ (Italpollina S.p.A., Rivoli Veronese, Italy) was used in the current greenhouse experiment. Trainer^®^ is a commercial biostimulant obtained through enzymatic hydrolysis of proteins from legume seeds; it contains 50 g kg^−1^ of N as free amino acids, and soluble peptides (Colla et al., 2017a; Rouphael et al., 2017b). The aminogram of the product was (g kg^−1^): Ala (12), Arg (18), Asp (34), Cys (3), Glu (54), Gly (12), His (8), Ile (13), Leu (22), Lys (18), Met (4), Phe (15), Pro (15), Thr (11), Trp (3), Tyr (11), and Val (14) (Rouphael et al., 2018).

The commercial biostimulant Trainer^®^ was applied in both foliar spray and substrate drench treatments at a concentration of 2.5 ml L^−1^. The Trainer^®^ concentration was adopted based on the company recommendations. The PH-treated plants were uniformly sprayed (foliar spray treatment) or applied at a rate of 30 ml per plant (substrate drench treatment) two times during the experiment on 16 and 23 June (9 and 16 days after transplanting, respectively). A 5-L stainless style sprayer “Vibi Sprayer” (Volpi, Piadena, Italy) was used in the foliar spray treatment. In both application dates, the PH treatments (foliar spray and substrate drench) were performed at 10:00 with an average air temperature inside the greenhouse of 24°C and relative humidity of 65%.

Nutrient solution was applied through the drip irrigation system and delivered at a rate of 2 L min^−1^. The composition of the basic nutrient solution used in the current study was: 32 mg L^−1^ S, 31 mg L^−1^ P, 117 mg L^−1^ K, 24 mg L^−1^ Mg, 1.12 mg L^−1^ Fe, 0.5 mg L^−1^ Mn, 19.0 μg L^−1^ Cu, 104.6 μg L^−1^ Zn, 216.0 μg L^−1^ B, and 28.8 μg L^−1^ Mo. The two N levels in the nutrient solution were obtained by adding calcium ammonium nitrate (14.2% nitrate and 1.3% ammonium) to the basic nutrient solution at 22.6 mg L^−1^ (7 mg L^−1^ N) or 720.0 mg L^−1^ (112 mg L^−1^ N). Moreover, in the low nitrogen solution, calcium chloride (CaCl_2_) was added at 831 mg L^−1^ to balance the calcium concentration (160 mg L^−1^) in both nutrient solutions.

### Biomass Production, Partitioning, and SPAD Index

On June 26 (19 days after transplanting; 72 h after the second biostimulant application), five plants per experimental unit were sampled and separated in leaves, stems and roots. All plant tissues were dried at 60°C for 72 h until they reach a constant weight to determine dry biomass production and partitioning. The number of leaves per plant was also counted.

On the same date, the soil plant analysis development (SPAD) index was measured on fully expanded leaves by means of a portable chlorophyll meter SPAD-502 (Konica Minolta, Japan). Ten healthy and fully expanded leaves were randomly measured and averaged to a single SPAD value for each experimental plot.

### Nitrogen Analysis

The dried leaf tissues, sampled from the first fully expanded leaves at 48 and 72 h after the second biostimulant application (18 and 19 days after transplanting) were ground in a Wiley Mill to pass through a 841 μm screen; then 1 g of dried leaf samples were analyzed for total nitrogen, nitrate, and ammonium.

Nitrogen (total N) concentration was assessed after mineralization with sulfuric acid (96%, Carlo Erba Reagents, Milan, Italy) in the presence of potassium sulfate and a low concentration of copper by the Kjeldahl method (Bremner, 1965).

Mineral N in the form of nitrate (N-NO_3_) and ammonium (N-NH_4_) was determined spectrophotometrically (Helios Beta Spectrophotometer, Thermo Electron Corporation, United Kingdom) using the salicylic-sulfuric acids and the salicylate-hypochlorite methods, respectively (Cataldo et al., 1975; Anderson and Ingram, 1989).

### Collection of Samples, RNA Extraction, and Purification

Two terminal leaflets were sampled from the first fully expanded leaves as well as fine roots of two plants per experimental plot at 6 h after the second biostimulant application, and immediately frozen in liquid nitrogen and stored at −80°C for molecular analysis. Samples of fresh leaves and roots were frozen and then grinded in liquid nitrogen.

Total RNA was isolated from homogenized leaf and root tissues according to the manufacturer’s instructions of the Spectrum Total Plant RNA Kit (Sigma-Aldrich, St. Louis, MO, United States) and re-suspended in 50 μl of DEPC-treated water. RNA concentration and quality were evaluated using a Multiskan GO Microplate Spectrophotometer (Thermo Fisher Scientific, Madison, WI, United States) and by agarose gel electrophoresis.

### Quantitative Real-Time PCR (qRT-PCR)

One microgram of the extracted RNA was used as template for the synthesis of cDNA, following the protocol of the QuantiTect Reverse Transcription Kit (Qiagen, Hilden, Germany). qRT-PCR was performed in a CFX 96 Real-Time PCR Detection System device (Bio-Rad, Hercules, CA, United States); each reaction was carried out in a volume of 15 μl, containing 7.5 μl of SsoAdvanced^TM^ SYBR^®^ Green supermix (Bio-Rad, Hercules, CA, United States), 1 μl of cDNA and 0.5 mM of each primer. qRT-PCR conditions were: an initial denaturation at 94°C for 30 s, followed by 40 cycles at 94°C for 5 s, 60°C for 30 s and melt curve analysis ranging from 65 to 95°C with 0.5°C per 5 s increments. Relative levels of transcript abundance were estimated as described in Sestili et al. (2010). Three biological samples per treatment were analyzed with three technical replicates per sample; each qRT-PCR data point represented the mean of three biological samples. A list of all genes analyzed throughout this study along with the corresponding primer pairs is provided in **Supplementary Table S1**.

### Statistical Analysis of Data

Analysis of variance of the experimental data set was assessed using SPSS 13 for Windows, 2001 (SPSS Inc., United States). To separate treatment means within each measured parameter, Duncan’s multiple-range test was performed at *P* ≤ 0.05.

## Results and Discussion

### Growth Responses and Nitrogen Concentration in Tomato Plants

In the current study, SPAD index (i.e., greenness readings), the dry weight of stems, root and total dry weight were influenced by N level in the nutrient solution and biostimulant treatments with no significant N level × biostimulant interaction, whereas the leaf number per plant and leaf dry weight were only affected by N regime (**Table 1**). When averaged over biostimulant application, a significant difference between the two N concentrations in the nutrient solution was recorded, with the highest values of leaf number, SPAD index, and total dry weight recorded at high N level (**Table 1**). Concerning the influence of the commercial legume-derived PH application on growth responses, the root and total dry weight as well as SPAD index were higher by 21, 17, and 7%, respectively, in substrate drench treatment in comparison to untreated tomato plants with no significant difference between the two modes of application (foliar spray and substrate drench; **Table 1**).

**Table 1:** Effect of nitrogen level in the nutrient solution and biostimulant mode of application on leaf number, soil plant analysis development (SPAD) index, dry weight of leaves, stems, roots, and total biomass of tomato plants at 19 days after transplanting.

Source of variance	Leaf number (no. plant^−1^)	SPAD index	Dry biomass (g plant^−1^)			
			Leaves	Stems	Root	Total
Nitrogen level	^∗∗∗^	^∗∗∗^	^∗∗∗^	^∗∗∗^	^∗∗∗^	^∗∗∗^
Biostimulant	*ns*	^∗∗^	ns	^∗∗∗^	^∗∗^	^∗∗^
Nitrogen level × Biostimulant	ns	ns	ns	ns	ns	ns
**Nitrogen level (mg L^−1^)**						
7	10.4 ± 0.3b	46.0 ± 0.5b	4.94 ± 0.20b	3.58 ± 0.12b	1.22 ± 0.06b	9.74 0.31b
112	11.9 ± 0.3a	56.0 ± 0.9a	7.76 ± 0.27a	6.36 ± 0.19a	2.04 ± 0.06a	16.15 ± 0.47a
**Biostimulant**						
No application	11.0 ± 0.3	49.5 ± 1.3b	6.02 ± 0.51	4.52 ± 0.40b	1.49 ± 0.15b	12.01 ± 1.02b
Foliar spray	11.3 ± 0.5	50.7 ± 1.9ab	6.33 ± 0.49	4.89 ± 0.46ab	1.60 ± 0.13ab	12.82 ± 1.03ab
Substrate drench	11.3 ± 0.5	52.8 ± 1.8a	6.71 ± 0.54	5.50 ± 0.48a	1.80 ± 0.13a	14.00 ± 1.12a
**Nitrogen level × Biostimulant**						
7 mg L^−1^ N without biostimulant	10.2 ± 0.3	45.7 ± 0.7	4.82 ± 0.20	3.07 ± 0.12	1.09 ± 0.07	9.10 ± 0.73
7 mg L^−1^ N with foliar spray	10.8 ± 0.5	44.6 ± 0.5	4.94 ± 0.60	3.70 ± 0.14	1.16 ± 0.11	9.67 ± 0.35
7 mg L^−1^ N with substrate drench	10.3 ± 0.7	47.7 ± 0.9	5.06 ± 0.14	3.97 ± 0.18	1.41 ± 0.09	10.44 ± 0.34
112 mg L^−1^ N without biostimulant	11.8 ± 0.4	53.3 ± 1.3	7.09 ± 0.44	5.97 ± 0.34	1.89 ± 0.12	14.92 ± 0.72
112 mg L^−1^ N with foliar spray	11.8 ± 0.3	56.8 ± 1.0	7.83 ± 0.49	6.09 ± 0.28	2.04 ± 0.09	15.96 ± 0.88
112 mg L^−1^ N with substrate drench	12.2 ± 0.7	57.9 ± 1.8	8.35 ± 0.40	7.03 ± 0.20	2.19 ± 0.08	17.57 ± 0.58

*ns, ^∗∗^,^∗∗∗^: non-significant or significant at P ≤ 0.01, and P ≤ 0.001, respectively. Different letters within each column indicate significant differences according to Duncan’s multiple-range test (P = 0.05). All data are expressed as mean ± standard error, *n* = 3.*

A presumed mode of action behind the stimulation of biomass production in response to substrate drench application of PH could involve the increased presence of *signaling molecules* such as small peptides which are typical compounds of PHs. The former elicitors in the commercial legume-derived PH which are easily perceived by both plant tissues (leaf and root) (Matsumiya and Kubo, 2011) may have generated a signal transduction pathway through modulation of endogenous phytohormone biosynthesis (Ryan et al., 2002; Cavani and Ciavatta, 2007; Ertani et al., 2017). Our results are consistent with the findings of several research groups (Ertani et al., 2009; Matsumiya and Kubo, 2011; Colla et al., 2014; Ugolini et al., 2015), who observed that foliar spray or substrate drench applications of plant-derived PHs exhibited auxin- and/or gibberellin-like activities as demonstrated by laboratory bioassays (Ertani et al., 2009; Colla et al., 2014), thus stimulating plant growth and yield.

Another putative mechanism behind the biostimulant activity of legume-derived PH on crop performance is the stimulation of the root system architecture in particular the increase in root hair length and density (Matsumiya and Kubo, 2011), which may improve N use efficiency, leading to an increase in total biomass when N is limiting plant growth. These findings are in line with previous studies testing the stimulation action of plant-derived PHs on root and shoot biomass (Ertani et al., 2009; Colla et al., 2014). For instance, Ertani et al. (2009) demonstrated that short-term application (48 h) of PHs derived from enzymatic hydrolysis of alfalfa plants (applied at 0.01, 0.1, or 1 mL L^−1^) elicited dose-dependent increase of root dry mass (from 20 to 42%) in corn compared to the untreated control. These results were also consistent with those of Colla et al. (2014) who reported that treating tomato cuttings with 6 ml L^−1^ of the legume-derived PH increased root density and length in comparison to untreated plants, inducing a “*nutrient acquisition response*” that favors N uptake and translocation.

Total N in leaf tissue was influenced by N level in the nutrient solution and biostimulant treatments with no significant N level × biostimulant interaction, whereas the mineral N in the form of nitrate (N-NO_3_, at 48 and 72 h after the second biostimulant application) and ammonium (N-NH_4_, at 48 h after the second biostimulant application) incurred significant N level × biostimulant interaction (**Table 2**). A significant correlation (*p* < 0.01) was also observed between SPAD index and total leaf N content (Pearson’s coefficient 0.961). No significant differences among biostimulant applications were observed for leaf nitrate content under low N regime. However, under high N level the highest nitrate concentration was observed with substrate drench (48 h) and with both foliar and root application (72 h) (**Table 2**). The positive effect of amino acids on the uptake and assimilation of nitrates under high N regime (70–140 g L^−1^) was previously described in other vegetable crops (radish and pepper) grown hydroponically (Liu and Lee, 2012). Our results also showed that the highest concentration of ammonium (at 48 h) was recorded with foliar spray application under high N regime (**Table 2**). Similarly to the effects on biomass production and partitioning, the total N as well as the nitrate and ammonium concentrations at high N level were significantly higher than those obtained from tomato plants grown at low N regime (**Table 2**). A different behavior was observed under low N regime with an increase of total N concentration after biostimulant application without any significant effect on nitrate concentration (**Table 2**). Because the only sources of nitrogen for plant uptake were the mineral fertilizer and the biostimulant, we hypothesized that the increase of the total N concentration in leaves of plants grown under low N fertilization regime may be due to the plant uptake of organic N (amino acids and peptides) coming from the biostimulant.

**Table 2:** Effect of nitrogen level in the nutrient solution and biostimulant mode of application on nitrate, ammonium, and total nitrogen of leaves in tomato plants.

Source of variance	N-NO_3_ (mg⋅kg^−1^ FW)		N-NH_4_ (mg⋅kg^−1^ FW)		Total N (g⋅kg^−1^ DW)
	48 h	72 h	48 h	72 h	
Nitrogen level	^∗∗∗^	^∗∗∗^	^∗^	^∗∗∗^	^∗∗∗^
Biostimulant	^∗∗^	^∗∗∗^	ns	ns	^∗∗^
Nitrogen level × Biostimulant	^∗^	^∗∗∗^	^∗^	ns	ns
**Nitrogen level (mg L^−1^)**					
7	78.0 ± 3.3b	68.2 ± 4.5b	23.5 ± 1.4b	21.8 ± 1.0b	14.3 ± 0.3b
112	135.8 ± 11.7a	196.7 ± 23.4a	31.6 ± 4.7a	36.9 ± 2.2a	38.3 ± 0.5a
**Biostimulant**					
No application	86.7 ± 7.9b	95.6 ± 7.5b	24.3 ± 1.8	28.3 ± 3.6	25.6 ± 3.8b
Foliar spray	104.5 ± 12.5b	149.3 ± 39.5a	33.5 ± 7.0	29.9 ± 3.3	26.0 ± 3.7ab
Substrate drench	129.5 ± 21.3a	152.3 ± 42.7a	24.9 ± 2.4	29.9 ± 4.9	27.2 ± 3.5a
**Nitrogen level × Biostimulant**					
7 mg L^−1^ N without biostimulant	69.3 ± 2.3d	82.3 ± 2.9bc	26.5 ± 2.1b	20.9 ± 2.2	13.3 ± 0.3
7 mg L^−1^ N with foliar spray	79.0 ± 4.0cd	63.1 ± 1.3c	21.2 ± 3.0b	23.1 ± 1.7	13.8 ± 0.3
7 mg L^−1^ N with substrate drench	85.6 ± 6.2cd	59.1 ± 9.1c	22.8 ± 1.6b	21.5 ± 2.0	15.8 ± 0.2
112 mg L^−1^ N without biostimulant	104.1 ± 2.6bc	108.9 ± 9.6b	22.0 ± 2.4b	35.7 ± 2.2	37.9 ± 1.0
112 mg L^−1^ N with foliar spray	129.9 ± 10.7b	235.5 ± 19.1a	45.7 ± 9.2a	36.8 ± 2.2	38.2 ± 0.9
112 mg L^−1^ N with substrate drench	173.4 ± 17.3a	245.6 ± 18.2a	27.0 ± 4.6b	38.3 ± 6.8	38.7 ± 0.7

*Nitrate and ammonium were measured on first fully expanded leaves at 18 (48 h after biostimulant application) and 19 days (72 h after biostimulant application) after transplanting, while leaf N content was determined at 19 days after transplanting. All data are expressed as mean ± standard error, n = 3. ns,^∗^,^∗∗^,^∗∗∗^: non-significant or significant at P ≤ 0.05, P ≤ 0.01, and P ≤ 0.001, respectively, Different letters within each column indicate significant differences according to Duncan’s multiple-range test (P = 0.05).*

Irrespective of the N level in the nutrient solution, our results showed that substrate drench application of legume-derived PH Trainer^®^ elicited significant increase (+6.2%) of total leaf N content compared to untreated plants, whereas foliar spray treatment exhibited intermediate values (**Table 2**). Our findings on the beneficial effect of legume-derived PH application were in agreement with those of Colla et al. (2013, 2014) who reported that the leaf or root application of commercial PH-biostimulant stimulated N metabolism and incurred significant increase in leaf N content in maize seedling and tomato plantlets grown under controlled environments amounting to 18 and 22%, respectively. Furthermore, the higher SPAD index values observed in tomato plants treated with PH-biostimulant (substrate drench) could be also considered a mechanism by which PH application can promote N use efficiency. In fact, SPAD index is widely considered as a key indicator of chlorophyll and N content which have been often associated with a better crop performance (Colla et al., 2017a; Ertani et al., 2017).

### Transcript Levels of Nitrate, Ammonium, and Amino Acids Transporters

The transcript levels of the key genes encoding for nitrate, ammonium, and amino acid transporters were investigated to provide novel insights on the effect of PHs either as signaling molecules or N source.

In higher plants two distinct systems of nitrate uptake were reported: the low-affinity transport system, responsible for uptake in presence of high nitrate concentration (>1 mM) and the HATS, involved in nitrate uptake in presence of low nitrate concentration (between 1 μM and 1 mM) (Little et al., 2005). In tomato, five nitrate transporter (*NRT*) genes inducible by nitrate were described: two *NRT1* and three *NRT2* (Ono et al., 2000; Hildebrandt et al., 2002). The expression of several *NRT2* genes was up-regulated by nitrogen starvation, suggesting a role of these transporters in the stimulation of the HATS for NO_3_^−^ (Forde, 2000; Williams and Miller, 2001; Remans et al., 2006).

The expression analysis was carried out on two genes encoding high-affinity nitrate transporters belonging to *NRT2* family: *NRT2.1* and *NRT2.3* (Remans et al., 2006; Fu et al., 2015). Remans et al. (2006) demonstrated that *NRT2.1* plays a key role in the coordination of the root development, acting on lateral root initiation under low nitrate regime; whereas *NRT2.3* is involved in nitrate uptake and long-distance transport from root to shoot (Fu et al., 2015).

**FIGURE 1:**
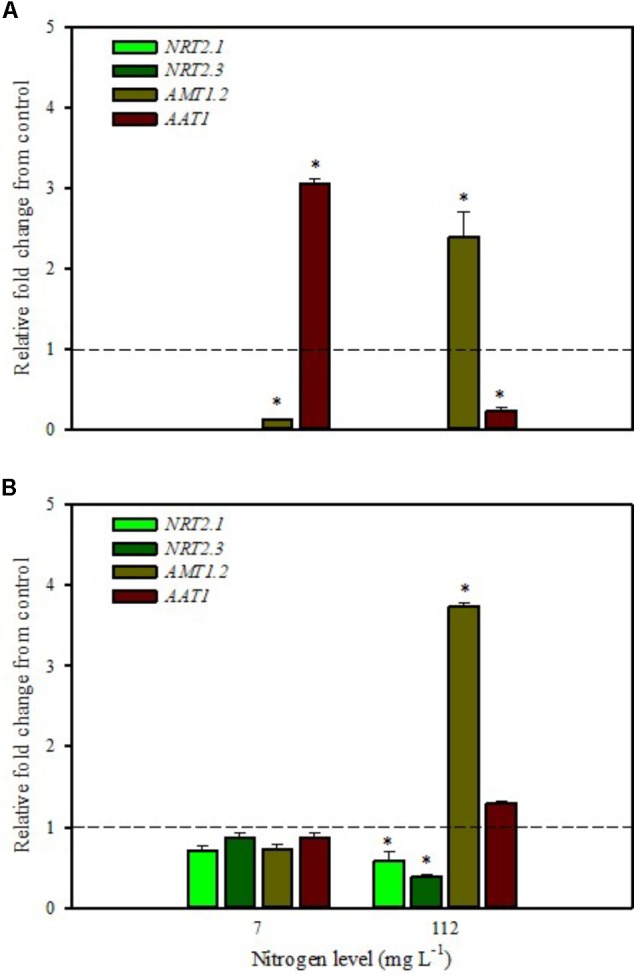
Gene expression of nitrate transporters (*NRT2.1* and *NRT2.3*), ammonium transporter (*AMT1.2*), and amino acid transporter (*AAT1*) in leaves **(A)** and roots **(B)** of tomato plants grown under low (7 mg L^−1^) and high nitrogen supply (112 mg L^−1^) after 6 h from foliar spray with a legume-derived protein hydrolysate. The values are reported as relative fold change from control, which was normalized to 1; values >1 represent up-regulation and <1 down-regulation. Vertical bars indicate ± standard error of means; ^∗^*P* < 0.05 compared with control.

**FIGURE 2:**
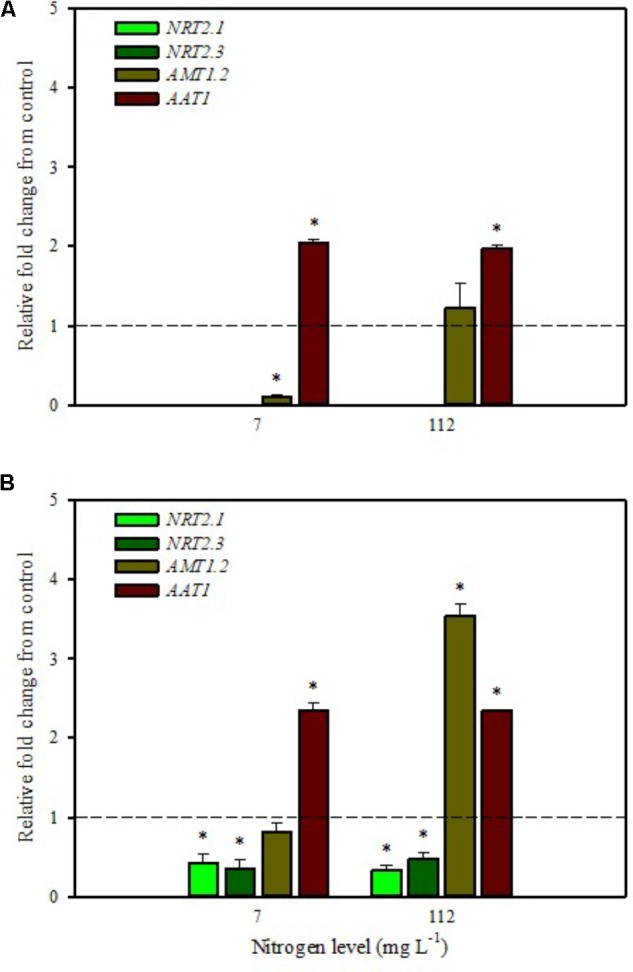
Gene expression of nitrate transporters (*NRT2.1* and *NRT2.3*), ammonium transporter (*AMT1.2*), and amino acid transporter (*AAT1*) in leaves **(A)** and roots **(B)** of tomato plants grown under low (7 mg L^−1^) and high nitrogen supply (112 mg L^−1^) after 6 h from substrate drench with a legume-derived protein hydrolysate. The values are reported as relative fold change from control, which was normalized to 1; values >1 represent up-regulation and <1 down-regulation. Vertical bars indicate ± standard error of means; ^∗^*P* < 0.05 compared with control.

Our analyses confirmed that these genes are only expressed in root and are undetected in leaves (**Figures 1A,B**, **2A,B**). Moreover, foliar applications of PH did not produce significant effects on the transcript levels of the genes encoding *NRT2.1* and *NRT2.3* in roots of tomato plants grown under low N level in the nutrient solution (**Figure 1B**). Conversely, both genes were drastically down-regulated at 6 h after the substrate drench application (**Figure 2B**); this different response of transcript levels between foliar and substrate drench application of PH may be due to the time needed by foliarly applied PH to reach the root system through the phloematic transport.

Little et al. (2005) reported that *NRT2.1* is involved in the repression of lateral root initiation under high sucrose/low nitrate growth conditions. The suppression of *NRT2.1* produced a phenotype able to initiate a large number of lateral roots in *Arabidopsis* (Malamy and Ryan, 2001). In the current study, the PH treated-tomato plants had a significant increase of root biomass (**Table 1**) compared to untreated ones; this phenotype can be correlated with the drastic repression of *NRT2.1* transcript. Transcript analyses of *NRT2.1* and *NRT2.3* in plants grown under high N concentration in the nutrient solution confirmed the repressor effect of the biostimulant (**Figures 1B**, **2B**). In this case both methods of PH applications (foliar spray and substrate drench) led to a drastic reduction of transcripts for both genes (**Figures 1B**, **2B**).

AMT ammonium transporters are integral membrane proteins that mediate the uptake of NH_4_^+^, a suitable nitrogen form for root uptake due to the reduced state of the nitrogen (Loqué and von Wirén, 2004). Although distinct *AMT* family members exist, we focused on *AMT1.2*, which encodes a high affinity transporter that is expressed in leaf and root tissue. This gene was strongly down-regulated after 6 h from foliar spray and substrate drench applications in leaves of tomato plants grown under low N supply (**Figures 1A**, **2A**).

The expression analysis, carried out on plants grown under high N conditions, showed a different regulation of the *AMT1.2* gene: it was strongly up-regulated in leaf and root at 6 h after foliar application of PH and only in root at 6 h after substrate drench treatment (**Figures 1A,B**, **2B**). The results suggested that both biostimulant application methods favored the ammonium translocation between apoplast and symplast cells under high N regime. von Wirén et al. (2000) demonstrated that the transcript level of *AMT1.2* was inducible by NH_4_^+^ and suggested that it could be involved in the retrieval of ammonium, thus compensating ammonium uptake from roots due to amino acid catabolism.

To elucidate the effects of PH application on amino acid turnover and allocation, the expression of a key gene encoding for an amino acid transporter was investigated. *AAT1* is a member of amino acid transporter family SL1.00sc07184_335.1.1, that is homologous to a member of the *Avt* family of vacuolar transporters belonging to the amino acid/auxin permease family isolated from *Saccharomyces cerevisiae* (*Avt1p*, GenBank No. NP_012534.1) (Snowden et al., 2015). The amino acid transporter encoded by *AAT1* is involved in the transport of glutamic acid, aspartic acid and isoleucine.

*AAT1* transcript was strongly induced in leaves (up to more than threefold) after foliar application of PH in plants grown under low N regime (**Figure 1A**); a positive regulation was also observed in roots and leaves after substrate drench application of PH (**Figures 2A,B**). No difference of *AAT1* transcript abundance was detected in root after foliar application of PH either under low or high nitrogen regime (**Figure 1B**). Our findings could be related to the time needed by foliarly applied PH to reach the roots through phloematic system. Furthermore, *AAT1* gene was strongly up-regulated after substrate drench application of PH in root and leaf tissues in tomato plants supplied with high N supply (**Figures 2A,B**), confirming an active role of this transporter in the amino acid allocation either in leaf or root tissues. However, a different behavior was observed after foliar application where the expression level of *AAT1* was markedly suppressed in leaf (**Figure 1A**). This different behavior could be associated to the different ability of leaf and root to uptake amino acid and peptides. Obviously, root cells could uptake peptides contained in the biostimulant product through permeases and hydrolyze them in amino acids; differently leaf cells have poor ability to uptake peptides or proteins.

### Transcript Levels of Key Genes Involved in Nitrogen Assimilation

**FIGURE 3:**
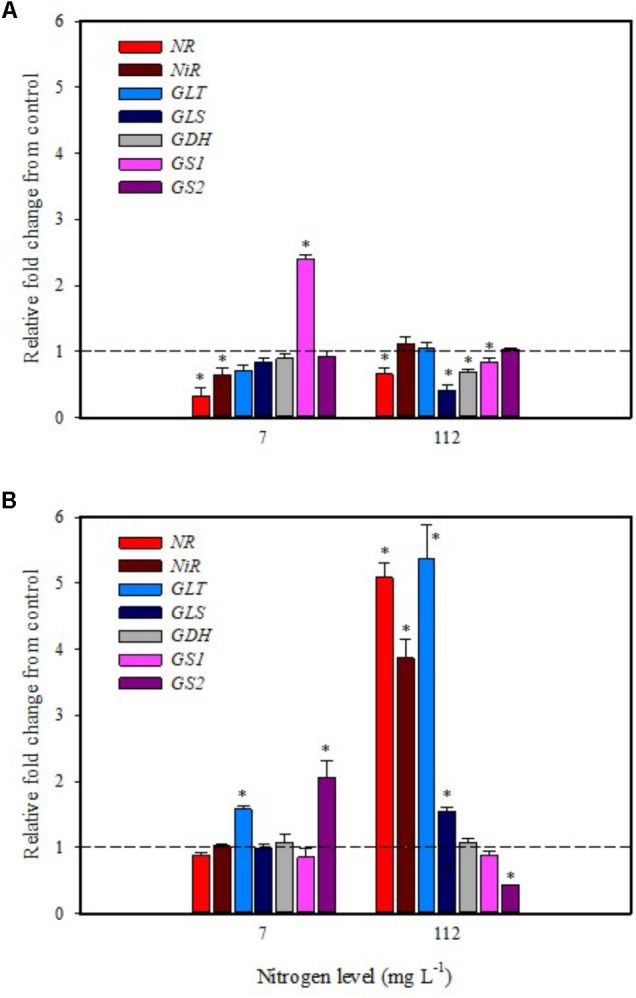
Gene expression of nitrate reductase (*NR*), nitrite reductase (*NiR*), ferredoxin-glutamate synthases (*GLT*), NADH-dependent glutamate synthases (*GLS*), glutamate dehydrogenase (*GDH*), and glutamine synthetase isozymes [cytosolic (*GS1*), and plastidic (*GS2*)] in leaves **(A)** and roots **(B)** of tomato plants grown under low (7 mg L^−1^) and high nitrogen supply (112 mg L^−1^) after 6 h from foliar spray with a legume-derived protein hydrolysate. The values are reported as relative fold change from control, which was normalized to 1; values >1 represent up-regulation and <1 down-regulation. Vertical bars indicate ± standard error of means; ^∗^*P* < 0.05 compared with control.

In the present study, expression data highlighted a drastic reduction of *NR* transcripts in leaves of tomato plants grown under low N regime after 6 h from foliar spray with PH (**Figure 3A**). The other genes (*NiR, GS2*, *GLT*, *GLS*, and *GDH*) were not affected except for *GS1* that was up-regulated twofold (**Figure 3A**). Foliar application of PH did not modulate the expression of *NR*, *NiR*, *GS1*, *GLS*, and *GDH* in root; only the genes *GLT* and *GS2* were up-regulated in comparison with untreated plants (**Figure 3B**).

**FIGURE 4:**
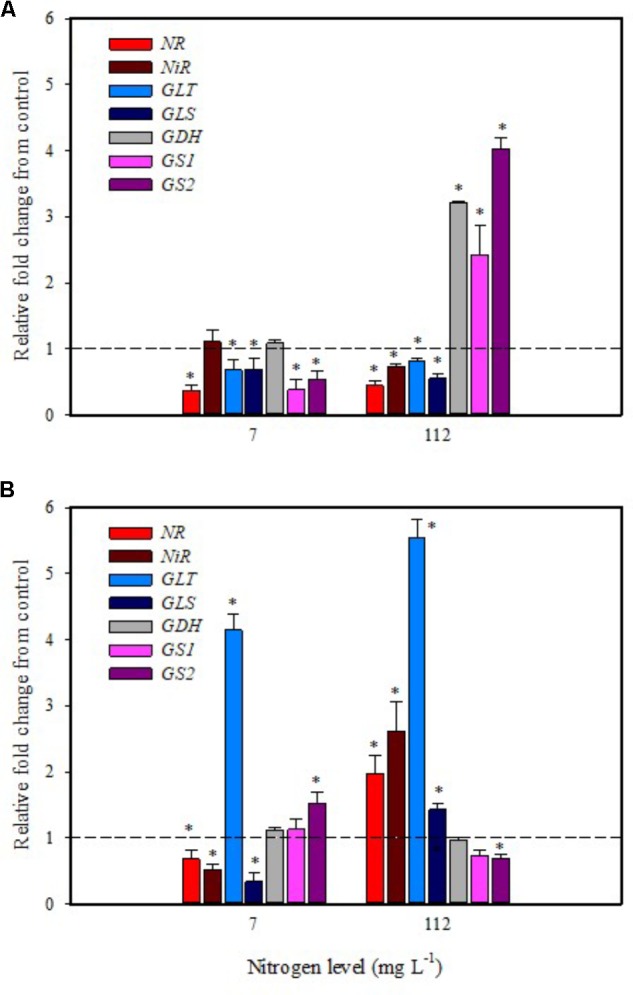
Gene expression of nitrate reductase (*NR*), *NiR*, ferredoxin-glutamate synthases (*GLT*), NADH-dependent glutamate synthases (*GLS*), glutamate dehydrogenase (*GDH*), and glutamine synthetase isozymes [cytosolic (*GS1*), and plastidic (*GS2*)] in leaves **(A)** and roots **(B)** of tomato plants grown under low (7 mg L^−1^) and high nitrogen supply (112 mg L^−1^) after 6 h from substrate drench with a legume-derived protein hydrolysate. The values are reported as relative fold change from control, which was normalized to 1; values >1 represent up-regulation and <1 down-regulation. Vertical bars indicate ± standard error of means; ^∗^*P* < 0.05 compared with control.

Substrate drench application of PH on tomato plants grown with low N availability had a remarkable repressive effect on the expression of *NR*, *GS1*, *GS2*, *GLT*, *GLS* in leaf and *NR*, *NiR*, *GLS* in root (**Figures 4A,B**). The remaining genes were not affected except for the *GLT* and *GS2* transcripts that were considerably increased in root (**Figure 4B**).

It was evident that both biostimulant application methods (foliar spray and substrate drench) down-regulated the key gene involved in the first steps of nitrate assimilation (*NR*) in leaves of plants grown under low N regime. These findings were associated with the reduction of transcript levels for the ammonium and nitrate transporters (**Figures 1A**, **2A**). Because of the increase in total biomass and total nitrogen content in PH treated plants (**Table 1**), we hypothesized that PH acted as N source especially when it was supplied as substrate drench treatment under low N regime. Plants can take up organic nitrogen compounds of low molecular mass, including amino acids and small peptides (di- and tripeptides), via membrane transporters (Paungfoo-Lonhienne et al., 2008). Moreover, roots exude proteolytic enzymes that digest large peptides leading to an increase of free amino acids for plant uptake (Paungfoo-Lonhienne et al., 2008). These findings may explain the better performances of Trainer^®^ in improving total N content of leaves when it was applied as substrate drench instead of foliar spray. This hypothesis was strengthened by the enhancement of *AAT1* expression both in leaves and roots after PH applications, suggesting a rapid mobilization of amino acids in plant tissues. For instance, Miller et al. (2007) suggested that amino acids (especially glutamine) can provide a signal for the regulation of nitrate uptake. In their study the authors found a strong reduction of the transcript levels of the nitrate and ammonium transporters in roots treated with exogenous amino acids. Another possible mechanism could be that PH mediated-root growth enhancement increased the root uptake of mineral nitrogen from the substrate especially under high N availability in the rootzone.

A different effect on the transcript levels of *NR*, *NiR*, *GS*, and *GOGAT* was observed in maize plants treated with an alfalfa protein hydrolysate (Schiavon et al., 2008) where these genes were significantly up-regulated by PH application. A plausible reason for the different behavior could be the growing conditions: Schiavon et al. (2008) provided the PH continuously for 48 h in a hydroponic system, whereas in our study plants were grown in substrate and treated twice with PH.

The application of legume-derived PH in tomato plants grown under high N concentration did not produce significant changes of transcript abundance for all genes in leaves at 6 h after foliar application (except for *NR, GDH, GLS* that were down-regulated) (**Figure 3A**); a different trend was observed in roots, where *NR*, *NiR*, *GLS*, and *GLT* were strongly induced and *GS2* was significantly repressed (**Figure 3B**). The application of legume-derived PH by substrate drench produced a similar effect on *NR*, *NiR*, *GLS*, and *GLT* in roots (**Figure 4B**). These findings are consistent with those of Liu and Lee (2012) who demonstrated that the application of mixed amino acids incurred significant increase in the enzymes activities (NR, NiR, and GS) as well as the assimilatory pathway.

Glutamine synthetase 1, *GS2*, and *GDH* transcripts were strongly up-regulated in leaf at 6 h after PH applications as substrate drench (**Figure 4A**); this behavior could reveal a signaling activity of the biostimulant on the activation of amino acid turnover and ammonium recycling. A positive correlation between GS activity and nitrogen assimilation was previously described in wheat (Kichey et al., 2007). Moreover, several studies clearly demonstrated that GS activity was also associated with improved productivity in rice, wheat, and maize (Martin et al., 2006; Kichey et al., 2007; Brauer et al., 2011).

Overall, significant findings in the current work concerning the action of PH applications include their negative impact on the gene expression of *NRT2.1* and *NRT2.3*, which in turn promoted the development of the root apparatus in PH-treated plants compared to the untreated control. Another significant effect of PH application is the stimulation of N assimilation through increased expression of the two key genes for NR and NIR in plants grown under high N supply. Additionally, both methods of biostimulant application (foliar spray and substrate drench) strongly stimulated gene expression of the amino acid transporter *AAT1*, indicating that some free amino acids may be directly absorbed by plant. Finally, the data presented in the current paper contribute significantly toward the advancement of knowledge concerning the effects of plant biostimulants on plant growth and N content.

## Conclusion

The continuous and increasing pressure on vegetable growers and horticultural professionals to boost crop performance and at the same time to limit the use of synthetic mineral fertilizers, represents a strong motivation for the research community to seek for alternative technologies able to ensure high productivity in a sustainable manner (i.e., by enhancing nutrient use efficiency). Tomato growth as well as key genes involved in N assimilation was assessed in a multifactorial approach accounting for the influence of PH-biostimulant treatments and N regimes. At both nitrogen regimes, the application of legume-derived PH especially as a substrate drench enhanced the tomato performance parameters and N content indicating the importance of the application method. The increase in plant biomass was associated to the stimulation of the root growth, thus inducing a “*nutrient acquisition response*” that favors N uptake and translocation. Our results also demonstrated that PH application differentially regulated in a N-dependent manner the expression of genes involved in nitrate, ammonium and amino acid transporters as well as the key genes involved in N metabolism. Under low nitrogen supply, PH upregulated the expression of genes encoding for amino acid transporter and ferredoxin-glutamate synthases, and *GS* in roots whereas expression of genes encoding for nitrate and ammonium transporters, and *NR* were downregulated especially in leaves. Under high nitrogen supply, PH upregulated the expression of genes encoding for ammonium and amino acid transporter especially in roots and *NR*, *NIR*, and ferredoxin-dependent glutamate synthase in roots whereas expression of genes encoding for nitrate transporter in roots, and *NR* in leaves were downregulated. These results highlighted the potential benefits of using legume PH in tomato production to increase growth and N-nutritional status of plants grown under both high and low nitrogen regimes. Overall, the PH mediated-increase of total N content in leaves can be explained by the stimulation of root growth and the upregulation of genes involved in the N assimilation.

## Author Contributions

FS wrote the first draft of the manuscript and followed the molecular analysis and data interpretation. YR wrote many parts of the manuscript and followed the agronomic measurements. MC wrote many parts of the manuscripts and followed the samplings and analysis of nitrogen, nitrate, and ammonium. AP made the molecular analysis of gene expression. PB was involved in writing part of the manuscript on molecular analysis and data interpretation. RC was involved in the implementation of the manuscript. GC provided the intellectual input, set up the experiment, and corrected the manuscript.

## Conflict of Interest Statement

The authors declare that the research was conducted in the absence of any commercial or financial relationships that could be construed as a potential conflict of interest.
