# Supplementary material for: Protein Hydrolysate Stimulates Growth in Tomato Coupled With N-Dependent Gene Expression Involved in N Assimilation
Source: Front Plant Sci. 2018 Aug 22;9:1233. doi: 10.3389/fpls.2018.01233 (PMC6113680; doi:10.3389/fpls.2018.01233)
Supplement: Supplementary file 1 [file Table_1.DOC]

**Supplementary Table S1.** List of all genes analyzed throughout this study along with the corresponding primer pair.

| Gene name | Accession number | Primer (5’-3’) | Size (bp) | References |
| --- | --- | --- | --- | --- |
| LeNTR2.1 | AF092655 | F: TCCAGTCAAGGGAACGGAAGAACA  R: ACCACGCTCTGATCGGCAATTT | 118 | Ruzicka et al., 2010 |
| LeNTR2.3 | AY038800 | F: AATGCATGGTGTTACTGGTAGAGAG  R: CTAATAATAGGGACTAAAGGGGCTG | 237 | Yao et al., 2008 |
| LeNR | X14060 | F: GGTTCATCACTCCCGTACCACTT  R: TCTGCTTCACCATATTCTGCTCT | 216 | Yao et al., 2008 |
| LeGS1 | AJ277561 | F: ACACGGAGAAGGAAGGAAAGGGAT  R: TCCGCGATCATAGAGGTCACAACA | 88 | Ruzicka et al., 2010 |
| LeGS2 | U15059 | F: AGCTTGTACCTTGTTGAGTTTCCCT  R: CACCTCAACTCCAAAGTAGTCTCATCTCT | 118 | Ruzicka et al., 2010 |
| LeGDH | TC191551 | F: GTTTACTGGCTTGGACCTCAGTTT  R: CACTTTGTCCATGGGCAGAACTTACA | 84 | Ruzicka et al., 2010 |
| LeGLS | TC207820 | F: GCACAACCATGACCACGGTCTAAT  R: CAACTGCTGAAGCACCACGAAAGA | 178 | Ruzicka et al., 2010 |
| LeGLT | BG627938 | F: GGGAGAGTTTGAGGGTAGGGAATA  R: CAAGCAAACTGGCACGCACATA | 96 | Ruzicka et al., 2010 |
| LeNIR | AW039265 | F: GGATTCATGGGATGCCTGACTAGA  R: TTCTCGTGGAACTGCACCAAAGT | 189 | Ruzicka et al., 2010 |
| LeAMT1.2 | X95098 | F: TTGTACCGCCGCTCTGACAACTTT  R: CATGGCTCAACAACTGCACAACCT | 132 | Ruzicka et al., 2010 |
| AAT1 | XM_004250012 | F: CCCTGTGGCTATGAGTTTAGAG  R: CGA TCA ACG CCA TCA CAA ATG | 149 | See current article |
| α-tubulin | TC115716 | F: TGAGGTCTTCTCACGCATTGACCA  R: AATCCTTCTCGAGGGCAGCAAGAT | 198 | Coker and Davies, 2003 |

**References**

Coker, J.S., and Davies, E., 2003 Selection of candidate housekeeping controls in tomato plants using EST data. BioTechniques 35(4), 740-748.

Ruzicka, D.R., Barrios-Masias, F.H., Hausmann, N.T., Jackson L.E., and Schachtman D.P., 2010. Tomato root transcriptome response to a nitrogen-enriched soil patch. BMC Plant Biol. 10:75. doi: 10.1186/1471-2229-10-75

Yao, J. Shi W.M., and Xu, W.F., 2008. Effects of salt stress on expression of nitrate transporter and assimilation-related genes in tomato roots. Russian Journal of Plant Physiology. 55(2), 232-240. https://doi.org/10.1134/S1021443708020106
